# Recent Advances in Modification Strategies and Functional Applications of Raw Lacquer: A Comprehensive Review

**DOI:** 10.3390/ma19122489

**Published:** 2026-06-10

**Authors:** Xiao Li, Yihua Qian, Xiaoyu Wu, Yunyao Zheng, Xinhao Feng, Xinyou Liu

**Affiliations:** 1 College of Furnishing and Industrial Design, Nanjing Forestry University, Nanjing 210037, China; lixiaoxyz@njfu.edu.cn (X.L.); 2411404109qyh@njfu.edu.cn (Y.Q.); xiaoyv@njfu.edu.cn (X.W.); 2411402229@njfu.edu.cn (Y.Z.); fengxinhao@njfu.edu.cn (X.F.); 2 Co-Innovation Center of Efficient Processing and Utilization of Forest Resources, Nanjing Forestry University, Nanjing 210037, China

**Keywords:** raw lacquer, urushiol, nanocomposite modification, functional coatings, smart materials

## Abstract

Raw lacquer, a natural polymer derived from the bast of lacquer trees (*Toxicodendron vernicifluum*), is renowned as the “King of Coatings” due to its exceptional film-forming properties, abrasion resistance, corrosion resistance, and biocompatibility. However, its inherent limitations—including stringent drying conditions, slow curing rates, deep coloration, and difficult application—have severely restricted its modernization and widespread adoption. This review systematically summarizes recent research advances in the modification and application of raw lacquer, focusing on four major modification strategies: (1) Nanocomposite modification—incorporating functional nanofillers such as Al_2_O_3_, cellulose nanofibrils (CNF), polydopamine (PDA) melanin-like nanoparticles, and SiO_2_ to significantly enhance film hardness, compactness, UV-aging resistance, and drying kinetics. (2) Chemical structure modification—employing molecular design strategies including aminoanthraquinone grafting, tung oil blending, water-based emulsification, and terpene/allyl group functionalization to improve hydrophobicity, flexibility, fast-drying properties, and achieve dual photo/oxygen curing. (3) Biomass synergistic composites—utilizing natural polymers such as chitosan and lignin, along with bio-inspired adhesion mechanisms (e.g., PDA), to confer advanced functionalities including antibacterial and antifouling properties. (4) Curing behavior regulation—precisely controlling drying kinetics through inorganic salt ion microenvironment engineering, nonionic surfactants, and salicylaldehyde Schiff base-based driers. Building upon these foundations, this review further expands on the emerging high-value applications of modified lacquer in preventive conservation of cultural heritage, advanced functional coatings (anti-corrosion, super-hydrophobicity, flame retardancy), biomedical materials (hemostasis, antibacterial activity, drug-controlled release, water treatment adsorption), and intelligent responsive flexible electronics. Finally, addressing challenges including weak fundamental research, bottlenecks in green industrialization, and lack of standardization, future development directions are proposed encompassing interdisciplinary innovation, sustainable modification strategies, integration of multifunctional intelligent systems, and big data-driven research paradigms, aiming to provide theoretical guidance and technical references for the high-value utilization and modernization of lacquer resources.

## 1. Introduction

Raw lacquer, a natural polymer material derived from the phloem of the lacquer tree (*Toxicodendron vernicifluum*), is renowned as the “king of coatings” for its excellent film-forming properties, high hardness, abrasion resistance, corrosion resistance, and biocompatibility [1,2,3]. Archaeological research indicates that the application of raw lacquer dates back to the Neolithic Age, approximately 8000 years ago. Ancient Chinese lacquerware, buried for millennia, still retains its lustrous appearance and well-preserved substrates, fully demonstrating the exceptional durability and protective performance of raw lacquer coatings [2]. The unique chemical structure and film-forming mechanism of this ancient material endow it with significant research value and application potential in the field of modern functional materials [4]. However, the modern application of raw lacquer has long been constrained by its inherent drawbacks: demanding drying conditions (requiring 20–30 °C and 70–90% relative humidity), slow drying rate, dark color, high viscosity leading to application difficulties, as well as insufficient alkali resistance and anti-UV aging performance [5]. These limitations severely restrict the promotion of raw lacquer in high-value-added fields such as industrial coatings, biomedical materials, and smart materials. Driven by the growing global demand for green and sustainable materials, as well as the strategic orientation of China’s “14th Five-Year Plan” for Industrial Green Development towards bio-based materials, raw lacquer, as a natural renewable resource, has recently regained widespread attention from both academia and industry.

From the perspective of current research, the modification of raw lacquer has evolved from single-property improvement to a stage of systematic innovation involving multidisciplinary intersections. Researchers worldwide have conducted extensive work and achieved significant progress around four major strategies: nanocomposite modification, chemical structure modification, biomass synergistic compositing, and curing behavior regulation. Nanocomposite modification, through the introduction of nanoparticles such as Al_2_O_3_, SiO_2_, cellulose nanofibers (CNF), and polydopamine (PDA), significantly enhances film hardness, density, and anti-UV aging performance. Notably, biomimetic PDA modification can reduce the UV aging gloss loss rate by over 60% [6,7,8,9]. Chemical modification focuses on the structural design of the urushiol molecule, employing strategies such as aminoanthraquinone grafting, tung oil blending, water-based emulsification, and allyl modification, effectively improving hydrophobicity, flexibility, and rapid drying, while achieving photo/oxygen dual curing [10,11,12,13,14,15]. Biomass synergistic compositing draws inspiration from natural antifouling and biomimetic adhesion mechanisms, combining natural polymers such as chitosan and lignin with raw lacquer to impart advanced functionalities like antibacterial and antifouling properties [8,16]. Curing behavior regulation achieves precise control over drying kinetics through inorganic salt ion microenvironment engineering, nonionic surfactants, and Schiff base driers [17].

Nevertheless, existing reviews often focus on summarizing a single modification strategy or application direction, lacking a systematic integration of the entire “structure-modification-performance-application” chain. In particular, in-depth analyses of multi-scale interface regulation mechanisms, nano-bio synergistic effects, and emerging smart application areas remain insufficient. Furthermore, advances in the identification technology of raw lacquer germplasm resources (e.g., combining HPLC with ELISA) provide a new scientific perspective for understanding the performance differences in raw lacquer from different sources and their genetic basis at the molecular level, facilitating more targeted performance improvement. To address these gaps, this paper aims to systematically review the latest progress in the research of raw lacquer modification and application over the past five years, with a focus on the following four aspects: (1) Systematically outlining the mechanisms and technological pathways of the four major modification strategies, with particular emphasis on the critical role of nano-bio synergistic modification in overcoming the performance bottlenecks of raw lacquer; (2) Deeply analyzing the interfacial structure and curing kinetics regulation mechanisms of raw lacquer emulsion systems, establishing theoretical support for the entire “emulsification-curing-film formation” process; (3) Proactively expanding the application boundaries of raw lacquer in emerging fields such as cultural heritage protection, advanced functional coatings, biomedical materials, and smart responsive systems; (4) Introducing perspectives from germplasm resources and molecular identification technologies, exploring the paradigm shift from “empirical material selection” to “molecular design.” By constructing a multi-dimensional “structure-interface-performance” analytical framework, this paper aims to provide systematic theoretical guidance and technical references for the high-value utilization and modern transformation of raw lacquer resources.

Literature search methodology. To ensure the comprehensiveness and reliability of this review, a systematic literature search was conducted using the Web of Science Core Collection and Scopus databases. The search covered the period from January 2000 to April 2025. The following keywords and their combinations were used: (“raw lacquer” OR “urushiol” OR “natural lacquer” OR “Toxicodendron vernicifluum”) AND (“modification” OR “nanocomposite” OR “chemical modification” OR “waterborne” OR “curing behavior”) AND (“application” OR “coating” OR “anticorrosion” OR “biomedical” OR “smart material”). Only peer-reviewed journal articles, review papers, and doctoral dissertations written in English or Chinese were included. References cited in the retrieved articles were also manually screened to identify additional relevant studies. This methodological framework enabled the selection of 54 core references that form the basis of the present review.

## 2. Structure and Function of Raw Lacquer

The outstanding performance of raw lacquer originates from its unique chemical composition and multi-scale structure. A deep understanding of the compositional characteristics, molecular structure, and their structure–property relationships with macroscopic properties provides the theoretical foundation for rational design and functional modification. This chapter systematically elucidates the structural features and functional characteristics of raw lacquer from three dimensions: chemical composition, molecular structure, and physicochemical properties.

### 2.1. Composition and Structure of Raw Lacquer

**Figure 1:**
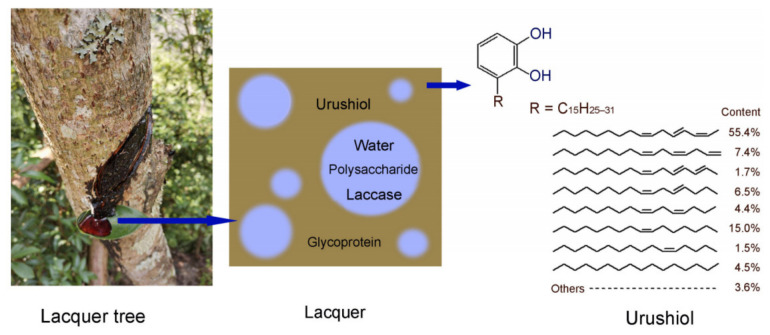
Composition of raw lacquer [22].

Raw lacquer is a natural water-in-oil (W/O) complex emulsion system, with its typical chemical composition including urushiol (60–75%), water (20–30%), lacquer polysaccharide (5–7%), laccase (<1%), and glycoproteins (<2%) (Figure 1). However, recent studies have demonstrated that these components are not simply mixed but form a colloidal system with multi-scale structure through specific interactions, and their spatial distribution and dynamic evolution have decisive effects on the film formation process [2,18,19,20].

Urushiol is the primary film-forming substance of raw lacquer, and its molecular structure consists of a catechol ring and a C15–C17 long side chain. The amphiphilic nature of urushiol—characterized by hydrophilic phenolic hydroxyl groups and a hydrophobic long alkyl chain—enables it to self-assemble into molecular films at the oil–water interface, which is crucial for maintaining emulsion stability [21]. The degree of unsaturation of the urushiol side chain (typically containing 1–3 double bonds) and its conjugation degree directly determine the activity of subsequent crosslinking reactions and the crosslinking density of the lacquer film.

Laccase, despite its extremely low content (<1%), serves as the core biocatalyst for the room-temperature curing of raw lacquer. Laccase is a copper-containing polyphenol oxidase whose active center catalyzes the oxidation of the catechol structure of urushiol to semiquinone radicals, thereby initiating polymerization [21]. The catalytic activity of laccase is highly dependent on environmental humidity, which explains the specific requirement of high humidity for raw lacquer drying.

Lacquer polysaccharide and glycoproteins play dual roles as structural stabilizers and interfacial regulators in raw lacquer. Lacquer polysaccharide forms a three-dimensional network structure that not only stabilizes the emulsion but also provides a suitable microenvironment for laccase; glycoproteins are involved in regulating the rheological properties and adhesion of the lacquer film [18].

It is noteworthy that a research team from Shandong University recently achieved the precise identification of four types of natural raw lacquer (Burmese lacquer, Rhus succedanea lacquer, large-pattern and small-pattern *Toxicodendron vernicifluum* lacquers) using a combination of high-performance liquid chromatography (HPLC) and enzyme-linked immunosorbent assay (ELISA) [23]. The study found that the small-pattern *Toxicodendron vernicifluum* lacquer exhibits a higher content of polar urushiol, and its glycoprotein molecular weight is significantly smaller than that of other varieties. Based on these differences, specific polyclonal antibodies were prepared, enabling clear recognition of raw lacquers from different sources and their mixtures. This method provides a reliable tool for archaeological identification and cultural heritage conservation, and also establishes a methodological foundation for constructing the correlation between “raw lacquer provenance–chemical composition–macroscopic performance”.

### 2.2. Molecular Structural Characteristics

**Figure 2:**
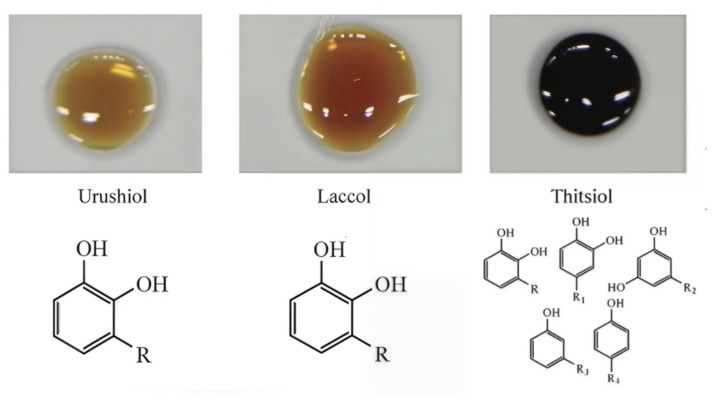
Chemical structures of urushiol, laccol, and thitsiol.

Depending on the species of the lacquer tree, the film-forming substances of raw lacquer can be classified into three main structural types: urushiol, laccol, and thitsiol [21]. The structural differences among these three types of compounds directly determine their polymerization behavior and the resulting performance of the lacquer film (Figure 2). Among them, urushiol is the primary component of raw lacquer produced by *Toxicodendron vernicifluum* in East Asia (China, Japan, and Korea). Its basic chemical structure consists of a catechol ring with a C15 straight-chain hydrocarbon group (pentadecyl) attached at the 3-position, and the side chain typically contains 1–3 non-conjugated double bonds (e.g., 8Z,11Z-pentadecadienyl group). This unsaturated side chain is not only the source of the lacquer film’s flexibility but also the key reactive site for auto-oxidative crosslinking reactions.

Laccol is mainly found in raw lacquer from *Toxicodendron succedanea* in Southeast Asia and the Taiwan region of China. Its structure is similar to that of urushiol, also being a catechol derivative, but its side chain is C17 (heptadecyl), which also contains 1–3 unsaturated double bonds. The longer C17 side chain, compared with C15, has a significant impact on the hydrophobicity and molecular packing mode of the lacquer film.

Thitsiol is derived from lacquer trees such as *Gluta usitata* in Myanmar, Thailand, and other regions, and exhibits the most complex structure. Its characteristics lie not only in the predominant use of a C17 side chain but also in the diversity of phenolic ring types and substitution patterns: in addition to the catechol structure, it contains a considerable proportion of resorcinol units; alkyl side chains can be simultaneously attached at the 3- and 4-positions of the phenolic ring, forming a disubstituted structure. This structural complexity results in significant differences in the polymerization behavior and lacquer film properties of thitsiol compared to East Asian lacquers [24,25].

**Figure 3:**
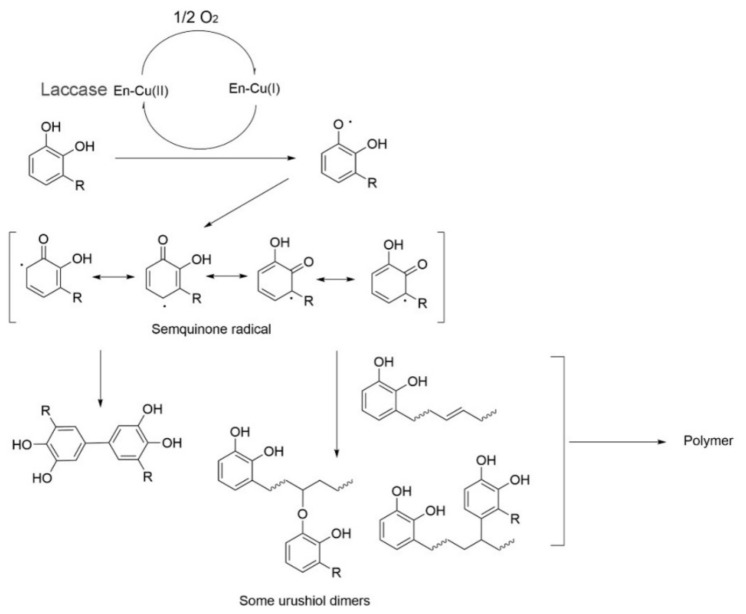
Chemical formulas of three urushiol homologs [22].

From the perspective of polymerization morphology, fresh raw lacquer is not a simple mixture of monomers. Harigaya et al., using gel permeation chromatography (GPC), found that a certain proportion of oligomers naturally exist in raw lacquer: monomers with an average relative molecular mass (Mr) of approximately 300 account for about 84%, while dimers (Mr ≈ 600) and trimers (Mr ≈ 1000) together account for approximately 16% [26]. These naturally occurring dimers exhibit diverse structures, including biphenyl-type and dibenzofuran-type structures formed by C-C bonds, as well as dimeric ether-type structures formed by C-O-C bonds (Figure 3). This finding reveals an important feature of the raw lacquer film formation process: the curing reaction does not start from scratch but proceeds via further crosslinking and network refinement based on pre-existing oligomers.

**Figure 4:**
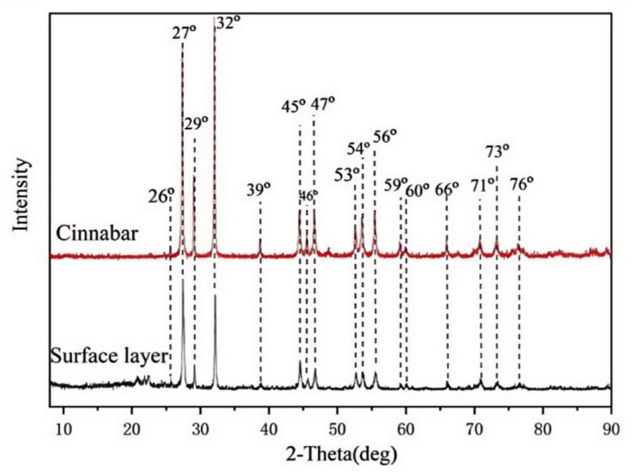
The XRD diffractogram of the surface layer and cinnabar [28].

To further elucidate the structural characteristics of solidified raw lacquer, Figure 4 presents a typical XRD profile of a cured lacquer film. The pattern exhibits a broad amorphous halo centered at approximately 2θ = 20°, indicating that the cured urushiol polymer network is predominantly amorphous in nature, lacking long-range crystalline order [27]. When the lacquer film contains crystalline additives (e.g., cinnabar pigment), additional sharp diffraction peaks corresponding to the specific crystalline phases (e.g., HgS) are superimposed on the amorphous background. This amorphous structure is responsible for the excellent flexibility and adhesion of raw lacquer films, while the dense crosslinked network provides high hardness and chemical resistance.

### 2.3. Performance of Raw Lacquer

#### 2.3.1. Mechanical Properties

The lacquer film formed after the curing of raw lacquer is renowned for its exceptional mechanical properties, and its hardness, adhesion, and flexibility can be finely tuned through chemical composition. Studies have shown that East Asian raw lacquers (e.g., from China, Japan, and Korea), which are primarily composed of urushiol, exhibit the best overall mechanical performance: the pencil hardness of pure urushiol-based lacquer films can reach as high as 9H, far superior to that of Vietnamese lacquers (pencil hardness below 1H) that are mainly composed of laccol [24]. This difference is mainly attributed to the polymerizable triene structure rich in the urushiol side chain, which can form a denser, more highly crosslinked three-dimensional network under the catalysis of laccase and auto-oxidation, thereby endowing the lacquer film with extremely high rigidity and scratch resistance [24,25].

In terms of adhesion, studies using the cross-hatch adhesion test to evaluate different lacquer films found that urushiol-containing lacquer films exhibit extremely strong adhesion to metal substrates (e.g., copper), achieving a rating of 4B (ASTM D3359 standard [29]), whereas the adhesion of pure laccol-based lacquer films drops sharply to the 1B level [24]. This indicates stronger interactions between urushiol-based polymers and metal surfaces, which is crucial for their application as anti-corrosion coatings. Nanoindentation tests further confirmed that Korean lacquer (predominantly urushiol) exhibits significantly higher nanoindentation hardness and modulus than Vietnamese lacquer (predominantly laccol) and Burmese lacquer (predominantly thitsiol) [25].

It is worth noting that mechanical properties can be tuned by blending different types of lacquers. For example, blending Korean lacquer with Burmese lacquer at a 50/50 (*w*/*w*) ratio resulted in a lacquer film whose drying speed and hardness were intermediate between the two, improving the slow drying and low hardness of Burmese lacquer while retaining some favorable characteristics, providing an effective approach for performance optimization [25]. However, improvements in mechanical properties often come with trade-offs in other properties: as the laccol content increases, the transparency and haze of the lacquer film improve, but mechanical hardness, adhesion, and corrosion resistance all show a decreasing trend [24]. This reveals the core contradiction in the design of lacquer materials-excellent mechanical properties resulting from high crosslinking density may come at the expense of optical properties.

#### 2.3.2. Hydrophobicity and Wettability

The intrinsic wettability of raw lacquer is weakly hydrophilic, with a water contact angle of approximately 78° for pure lacquer films [30]. However, precise control of surface micro-morphology can significantly alter its wetting behavior. Studies have shown that micro-/submicron-scale composite porous structures can be constructed on the surface of raw lacquer using a particle template method: when using 3000-mesh calcium carbonate particles as a template, a uniformly distributed hierarchical porous structure forms on the surface of the modified lacquer film, with the solid–liquid contact area fraction f1 reduced to approximately 8.7%, while the trapped air forming an “air cushion” accounts for as much as 91.3%. According to Cassie’s wetting theory, this rough structure increases the apparent contact angle to 153.5°, achieving super-hydrophobicity [30]. This result demonstrates that for materials with intrinsically weak hydrophobicity, surface micro-morphology design is an effective approach to obtain stable superhydrophobic properties.

#### 2.3.3. Anti-Corrosion and Durability

The exceptional anti-corrosion performance of raw lacquer has been directly validated by archaeological discoveries. Karpova E et al. conducted a study on Han Dynasty lacquerware unearthed from the Xiongnu tombs in Noin-Ula, Mongolia. Their research shows that although these lacquerware items were buried underground for nearly two millennia and subjected to complex soil environmental erosion, the main body of the lacquer films remained effectively preserved [31]. The most typical evidence comes from a lacquered wooden chariot component excavated from Tomb No. 22: the wooden substrate, found at a depth of 9–10 m below the surface, had almost completely decayed and disappeared, leaving only intact lacquer coating residues that clearly outline the original shapes of components such as umbrella ribs and wheel spokes. FTIR and Py-GC/MS analyses indicated that the main organic components of the lacquer film were urushiol and drying oil (possibly tung oil), which formed a dense, crosslinked polymer network through oxidative polymerization. This structure not only physically isolates water and microorganisms, but the chemical components themselves also possess antimicrobial and anti-mildew properties. In terms of weather resistance, raw lacquer exhibits excellent resistance to ultraviolet (UV) aging. Accelerated aging tests showed that after continuous irradiation for 1000 h in the 315–400 nm (UVA) band, the surface of the raw lacquer film developed only extremely fine cracks with widths less than 1 μm; under the same conditions, the crack widths of commercial synthetic polyurethane coatings generally exceeded 5 μm, accompanied by phenomena such as gloss loss and chalking [32]. This advantage stems from the partial absorption of UV light by the aromatic ring structure of urushiol and the inhibition of photo-oxidation reactions by the dense network.

#### 2.3.4. Antimicrobial Properties

**Figure 5:**
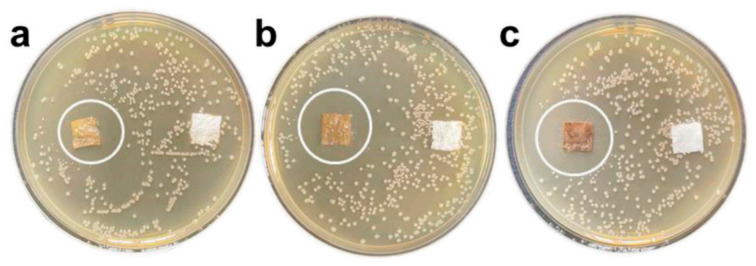
Antibacterial zone diagram of composite antibacterial coating on *E. coli*. The mass fractions of GZC antibacterial powder are 1% (**a**), 3% (**b**), and 5% (**c**) [35].

A study by Hyeon et al. [33] on urushiol-modified waterborne polyurethane-urea (PUU) composite films provides direct experimental data supporting the antimicrobial properties of raw lacquer. Antimicrobial tests conducted according to the JIS Z 2801 standard [34] over 24 h showed that the pure PUU film exhibited no significant antimicrobial activity against *Staphylococcus aureus* and *Escherichia coli* (R values of 0.0 and 0.7, respectively). When the urushiol content was increased to 22.2 wt%, the composite film exhibited an antimicrobial activity value (R) of 2.3 (99.4% inhibition rate) against *S. aureus* and an R value of 1.6 (97.7% inhibition rate) against *E. coli*, demonstrating excellent broad-spectrum antimicrobial capability (Figure 5). The researchers pointed out that urushiol exhibits particularly significant inhibitory effects against Gram-positive bacteria, which may be related to its mechanism of disrupting the cell wall.

### 2.4. Drying Mechanism

**Figure 6:**
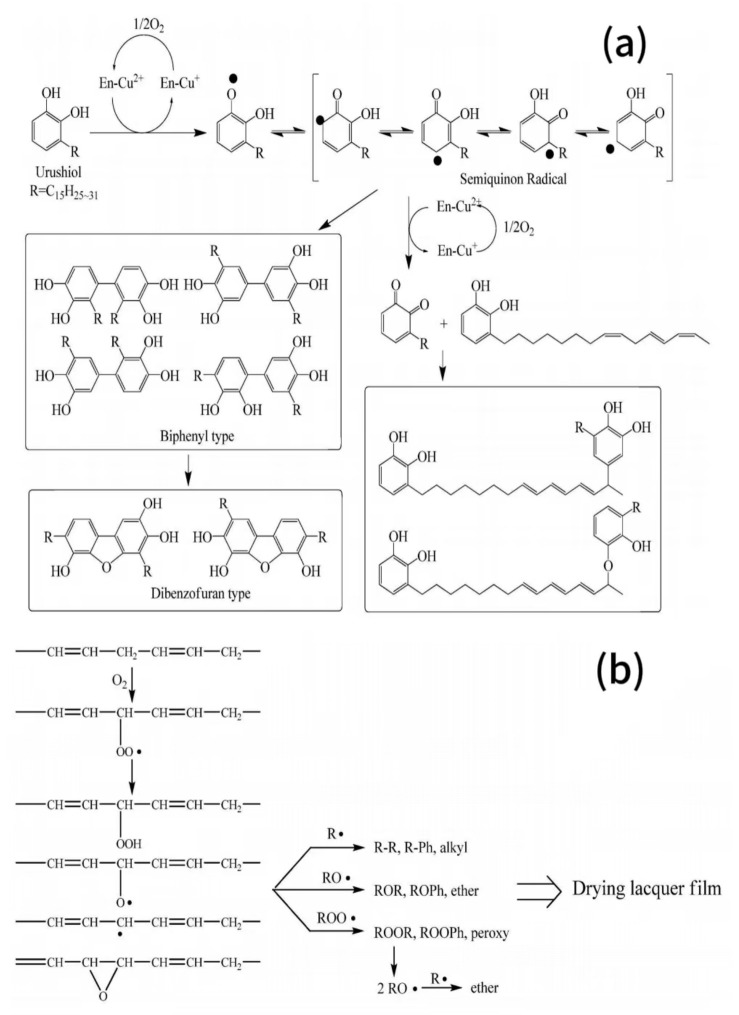
Polymerization reaction of urushiol [36].

The drying of raw lacquer is essentially a complex biocatalytic oxidative polymerization reaction, rather than a simple physical volatilization process. Its successful progression is highly dependent on specific environmental conditions—typically requiring a relative humidity of 70–80% and a temperature range of 20–30 °C [36]. The necessity of this high-humidity environment is reflected in two aspects: on the one hand, water is an essential medium for maintaining the three-dimensional conformation and catalytic activity of laccase; on the other hand, water molecules facilitate the formation of a hydrated oxygen layer on the surface of the lacquer film, promoting the diffusion of oxygen into the film interior, thereby sustaining the continuous redox cycle. The drying process can be divided into two stages (Figure 6):

Stage 1: Enzyme-catalyzed oxidative polymerization (5–8 h)

Laccase uses atmospheric oxygen to oxidize the catechol structure of urushiol into highly reactive semiquinone radicals, while itself being reduced to En-Cu^+^; subsequently, the semiquinone radicals undergo coupling reactions via C-C or C-O bonds to form dimers, trimers, and an oligomeric network. The reduced laccase is then re-oxidized by environmental oxygen, restoring its catalytic capability and forming an efficient catalytic cycle. At the end of this stage, the lacquer film reaches a “tack-free dry” state, where the surface is no longer sticky, but the interior is far from fully cured [36].

Stage 2: Auto-oxidative crosslinking (weeks to months)

As the unsaturated double bonds on the side chains of urushiol molecules become exposed, they begin to undergo non-enzymatic auto-oxidation reactions directly with oxygen, generating peroxide radicals, which further initiate chain crosslinking, forming a denser three-dimensional network structure. Although this process proceeds without enzyme involvement, it is key to the lacquer film ultimately achieving high hardness, excellent chemical resistance, and exceptional durability. Monitoring using the rigid pendulum test (RPT) shows that even 90 days after tack-free drying, the glass transition temperature (Tg) of the lacquer film continues to shift toward higher temperatures, fully demonstrating that crosslinking reactions proceed slowly over a long period [36].

Therefore, the “full curing” of raw lacquer is not a matter of days but rather a dynamic refinement process that can last for weeks or even months, ultimately forming a natural polymer material that combines high mechanical strength, chemical inertness, and biological stability. This unique stepwise curing mechanism is both the source of the excellent performance of raw lacquer and the fundamental reason for its demanding drying conditions and long curing duration.

## 3. Modification Strategies of Raw Lacquer

Although natural raw lacquer possesses excellent adhesion, abrasion resistance, solvent resistance, and biocompatibility, its inherent drawbacks—such as demanding drying conditions, long drying time, dark color, poor alkali resistance, susceptibility to photoaging, and difficulty in waterborne processing—severely limit its modern applications [16]. To overcome these bottlenecks, researchers have developed various modification strategies from multiple perspectives, mainly including nanocomposite modification, chemical structure modification, biomass synergistic composite modification, and curing behavior regulation, significantly expanding the application prospects of raw lacquer in the field of modern functional coatings.

### 3.1. Nanocomposite Modification

Nanocomposite modification involves uniformly dispersing functional nanoparticles into the raw lacquer matrix. Leveraging their high specific surface area and interfacial effects, this approach effectively enhances the mechanical properties, densification, thermal stability, and anti-aging ability of the lacquer film. Depending on the type of nanofiller, this can be further divided into inorganic nanoparticles and bio-based nanoparticles.

#### Inorganic Nanoparticle Modification

(1) Al_2_O_3_ Modification

**Figure 7:**
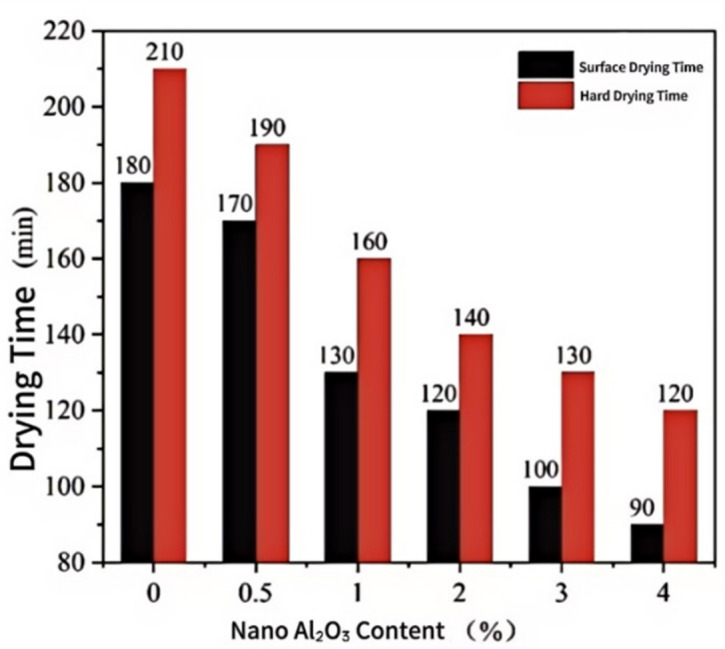
Drying time of raw lacquer and modified nano-Al_2_O_3_/raw lacquer composite lacquer films [6].

Alumina (Al_2_O_3_), as an inorganic nanomaterial with high hardness, high thermal stability, and good chemical inertness, is widely used in the functional modification of polymer matrix composites. Hanxing Wang et al. employed γ-aminopropyltriethoxysilane (KH550) to modify the surface of nano-Al_2_O_3_ and introduced it into the raw lacquer system through high-shear and ultrasound-assisted dispersion [6]. The study showed that after KH550 treatment, organic amino functional groups were grafted onto the surface of Al_2_O_3_, significantly reducing its surface polarity and improving its dispersion stability in raw lacquer. When the modified Al_2_O_3_ content was 1.0 wt%, the composite lacquer film exhibited optimal comprehensive performance: the pencil hardness increased from 3H for unmodified raw lacquer to 6H; the impact strength increased from 40 kg·cm to 50 kg·cm; and the surface drying time was shortened by approximately 28% (Figure 7). The strengthening mechanism mainly derives from two aspects: first, nano-Al_2_O_3_ acts as a rigid filler, providing a “skeleton” support during the curing process of the lacquer film, effectively restricting the movement of urushiol molecular chains; second, the active groups on the surface of the nanoparticles may participate in or catalyze the oxidative polymerization of urushiol, promoting densification of the crosslinking network. In addition, Al_2_O_3_ nanoparticles can effectively fill the micro-pores formed by curing shrinkage of the lacquer film, reducing the water and oxygen permeation rate. However, excessive addition of Al_2_O_3_ (>1.0 wt%) leads to nanoparticle agglomeration, forming stress concentration points, which instead reduces the flexibility and adhesion of the lacquer film.

(2) SiO_2_ Modification

Silica (SiO_2_) nanoparticles have become one of the most commonly used inorganic nanofillers in polymer coatings due to their advantages of high transparency, good chemical stability, and low cost. Pan Haiyun et al. [9] systematically investigated the effects of different contents (0–1.0 wt%) of nano-SiO_2_ on the performance of raw lacquer films. The results showed that within the range of 0–0.50 wt%, SiO_2_ could be uniformly dispersed in the raw lacquer matrix. At 0.25 wt%, the pencil hardness increased from 3H to 4H; at 0.50 wt%, the hardness remained at 4H, and the adhesion was rated as Grade 1 in both cases. However, when the addition exceeded 0.50 wt%, nanoparticle agglomeration occurred, leading to a rough lacquer film surface, a decrease in adhesion to Grade 2, and a reduction in hardness back to 3H (Table 1). Meanwhile, the glossiness of the lacquer film gradually decreased with increasing SiO_2_ content, dropping from approximately 70% for pure raw lacquer to about 40% at 1.00 wt% (approaching a matte effect). The strengthening mechanism of SiO_2_ mainly originates from its rigid support effect, acting as an inorganic hard core to provide physical reinforcement within the urushiol crosslinking network. This modification strategy is suitable for application scenarios that require high surface hardness and wear resistance, with acceptable moderate reduction in gloss.

(3) Cellulose Nanofibrils (CNF) Modification

Cellulose nanofibrils (CNF) are one-dimensional nanomaterials extracted from natural cellulose, possessing a high specific surface area (approximately 10,000 cm^2^/g), high Young’s modulus (100–140 GPa), a high aspect ratio (approximately 70), and fully renewable green characteristics. Wang Hanxing used KH550 to hydrophobically modify CNF to improve its dispersion and interfacial compatibility in raw lacquer [7]. The study showed that at a CNF addition of 10 wt%, the composite lacquer film exhibited optimal comprehensive mechanical properties: pencil hardness increased from 3H for pure raw lacquer to 5H, impact strength increased from 40 kg·cm to 48 kg·cm, and adhesion improved from Grade 5 to Grade 3. The drying time was significantly shortened with increasing CNF content; at a CNF content of 20 wt%, the through-drying time was reduced from 210 min for pure raw lacquer to 40 min, a reduction of 81%. The addition of CNF gradually decreased the water contact angle of the composite lacquer film from 87.57° for pure raw lacquer to 81.87° (at 20 wt% CNF), indicating a slight increase in surface hydrophilicity. However, the composite coating still exhibited excellent moisture resistance on wood surfaces: in moisture absorption cycling tests, wood coated with 10% CNF-modified raw lacquer showed the lowest tangential, radial, and volumetric swelling rates, demonstrating better dimensional stability than wood coated with pure raw lacquer or Al_2_O_3_-modified raw lacquer. This effect is mainly attributed to three factors: first, CNF forms a dense and uniform three-dimensional network structure within the lacquer film, filling the film pores; second, CNF undergoes esterification reactions and hydrogen bonding with urushiol, promoting crosslinking polymerization; third, CNF tightly crosslinks with cellulose and hemicellulose in the wood cell wall through chemical bonds and hydrogen bonds, and its rigid structure restrains the hygroscopic swelling of wood microfibrils.

However, in terms of UV aging resistance, the improvement achieved by CNF-modified raw lacquer was limited: after 480 h of artificial accelerated UV aging, the total color difference (ΔE*) of wood coated with 10% CNF-modified raw lacquer was 21.51, slightly lower than that of wood coated with pure raw lacquer (22.39), whereas the total color difference in wood coated with 1% Al_2_O_3_-modified raw lacquer was only 11.88. Therefore, the main advantages of CNF-modified raw lacquer lie in improving the film densification, mechanical properties, and wood dimensional stability, making it suitable for indoor wood coatings with high requirements for environmental friendliness and moisture resistance.

(4) Melanin-like Nanoparticles (PDA) Modification

Melanin-like nanoparticles generally refer to polydopamine (PDA) nanoparticles formed by the self-oxidative polymerization of dopamine under weakly alkaline conditions. PDA exhibits excellent adhesion, free radical scavenging ability, metal ion chelating properties, and broad-spectrum UV absorption, and has been widely used in recent years in the fields of biomaterials, anti-corrosion coatings, and anti-aging applications. Renjin Gao et al. [8] first introduced PDA nanoparticles into the raw lacquer system and systematically investigated their effects on the performance of lacquer films. PDA nanoparticles with a particle size of approximately 200 nm were prepared by self-oxidative polymerization of dopamine hydrochloride under weakly alkaline conditions (pH 8.5–9.0) for 24 h, and then dispersed into raw lacquer by stirring blending. The results showed that at a PDA addition of 4 wt%, the modified lacquer film exhibited optimal comprehensive performance: no change was observed after immersion in 10% NaOH solution for 1 day (pure raw lacquer film blistered within 1 day), indicating significantly enhanced alkali resistance. TGA tests showed that the char residue increased with increasing PDA content, reaching a maximum residual amount of 33%, indicating improved thermal stability. The most prominent improvement was in UV aging resistance—after 240 h of UV irradiation, the gloss loss rate of the modified lacquer film containing 4% PDA was only 9.2%, much lower than that of unmodified raw lacquer (22.8%), with no obvious surface powdering or cracking. Mechanistic studies indicate that PDA molecules contain a large number of phenolic hydroxyl groups, amino groups, and other functional groups, which can form a three-dimensional interpenetrating network structure and hydrogen bonding with urushiol, while also filling film pores and improving densification, thereby effectively blocking the erosion of alkaline media and UV light. Although PDA is dark brown and darkens the lacquer film color, its excellent weatherability and chemical resistance make it a highly valuable modifier in application scenarios where light colors are not critical (such as marine structures and outdoor wooden architectures).

In addition to its role as a nanofiller, PDA has also been employed as a bio-inspired adhesion enhancer in other modification strategies. Its catechol-rich structure, mimicking mussel foot proteins, enables strong interfacial bonding and has been utilized in various composite systems to improve coating adhesion and durability. These complementary applications of PDA are discussed in the context of biomass synergistic composite modification.

### 3.2. Physicochemical Modification

Chemical modification involves molecular design of urushiol, the primary film-forming substance of raw lacquer, or the introduction of functional groups to regulate its reactivity and film-forming performance from the ground up. According to the modification pathway, this can be subdivided into two categories: molecular grafting functionalization and waterborne modification.

#### 3.2.1. Molecular Grafting Modification

(1) Aminoanthraquinone Grafting

Huang Weiqi et al. [10] employed 2-aminoanthraquinone to graft modify urushiol, synthesizing a 2-aminoanthraquinone-urushiol modified coating film through a two-step method. First, urushiol was reacted with chloroacetyl chloride to prepare the urushiol chloroacetyl chloride derivative; subsequently, under the catalysis of potassium iodide/potassium carbonate, the amino group of 2-aminoanthraquinone underwent a nucleophilic substitution reaction with the chloroacetyl group, forming a –CH_2_CO–NH– linkage structure to obtain the 2-aminoanthraquinone-urushiol modified coating film. Performance tests showed (Table 2) that the surface drying time of the modified lacquer film was shortened from 240 min for pure raw lacquer to 140 min; pencil hardness increased from H grade to 6H; impact strength increased significantly from 18 cm to 56 cm; flexibility improved from 16 mm to 4 mm; and adhesion grade improved from Grade 5 to Grade 3. Particularly noteworthy was the increase in water contact angle from 70.9° to 105.7°, transforming from hydrophilic to hydrophobic. After immersion in 10% H_2_SO_4_, 10% NaOH, and 10% NaCl solutions for 7 days, the modified lacquer film showed no blistering, peeling, or obvious discoloration. The initial thermal decomposition temperature increased from 192 °C to 273 °C, indicating significantly enhanced thermal stability. SEM analysis revealed an irregular wrinkled structure on the surface of the modified film, forming a three-dimensional interpenetrating network structure that effectively blocked the penetration of corrosive media. This work not only addressed traditional issues of raw lacquer such as slow drying, low hardness, and poor alkali resistance, but also achieved its hydrophobic functionalization, opening new pathways for the application of raw lacquer in fields such as lacquer painting, crafts, and architectural decoration.

(2) Terpene-Based Composite Modification

Xiangling Sun et al. combined natural raw lacquer with a one-component self-emulsifying terpene-based waterborne polyurethane (TUWPU) to construct a novel blended emulsion system [14]. Terpene-based polyols were synthesized from β-pinene through hydrogenation, oxidative ring-opening, and other steps, and then reacted with hydrophilically modified HDI trimer to prepare the TUWPU prepolymer. Raw lacquer was directly added to the TUWPU emulsion, and under the synergistic emulsification effect of the hydrophilic segments of the terpene-based polyurethane and the lacquer polysaccharide in raw lacquer, a homogeneous, non-layered blended system was formed. FT-IR analysis showed that no chemical reaction occurred between raw lacquer and TUWPU, and compatibility was mainly achieved through physical emulsification. In terms of performance, the water absorption rate of the composite film decreased with increasing raw lacquer content. The glass transition temperature (Tg) increased from 28.34 °C for pure TUWPU to 39.84 °C (at 20% raw lacquer content), with a tensile strength of 11.04 MPa and an elongation at break of 68.27%. This strategy cleverly utilizes terpene-based polyurethane as a “green carrier” to achieve stable dispersion of raw lacquer in an aqueous system, providing a new approach for the development of fully biomass-based waterborne coatings.

(3) Allyl Modification

**Figure 8:**
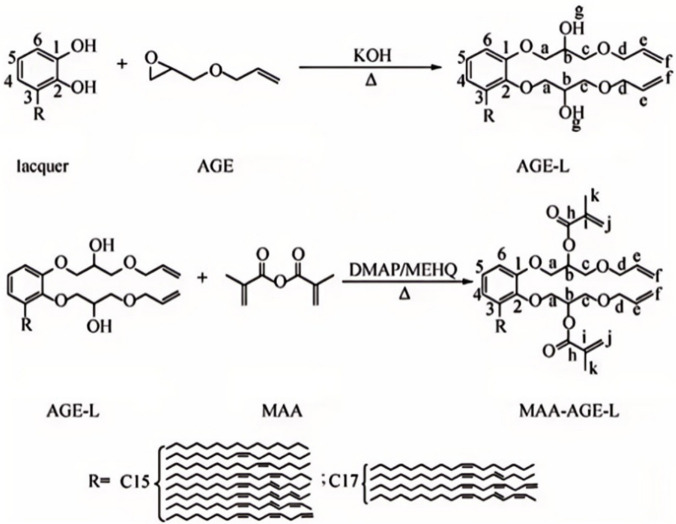
Synthesis route of allyl-modified raw lacquer [15].

To overcome the limitation that raw lacquer curing relies on a high-humidity environment and can only be catalyzed by laccase, Jian Chen et al. modified raw lacquer using allyl glycidyl ether (AGE) and methacrylic anhydride (MAA). They synthesized two types of allyl-modified products—allyl ether-modified raw lacquer (AGE-L) and allyl ether ester-modified raw lacquer (MAA-AGE-L)—and investigated their photo/oxygen curing characteristics [15]. The synthesis route is shown in Figure 8.

Curing experiments showed that in the presence of 3% cobalt isooctanoate drier, the surface drying time of MAA-AGE-L in oxygen at 30 °C and 80% relative humidity was shortened to 50 min, compared with 2 h for raw lacquer. Under UV light irradiation (365 nm, 2160 W/m^2^) with the addition of 3% photoinitiator 1173, the surface drying times of MAA-AGE-L and AGE-L were shortened to 10 s and 30 s, respectively, much faster than that of unmodified raw lacquer (50 s), which is attributed to the differences in curing reactivity of the allyl ester and allyl ether double bonds. The pencil hardness of both AGE-L and MAA-AGE-L after curing reached 2H. AGE-L exhibited an adhesion grade of 1 after UV curing, while MAA-AGE-L showed relatively poorer adhesion (Grade 5 for UV curing, Grade 3 for oxygen curing). Both modified products exhibited good flexibility, impact strength, and resistance to water, ethanol, saline solution, and acids, but insufficient alkali resistance. This study achieved efficient photo/oxygen dual curing of raw lacquer derivatives under non-enzymatic conditions, eliminating the dependence on high-humidity environments and laccase activity. Moreover, the color of the modified lacquer film was lightened, greatly expanding its application scenarios [15].

#### 3.2.2. Waterborne Modification

Waterborne modification is a key breakthrough for the modern application of raw lacquer. Zheng Yanyu addressed and overcame the technical challenges of rendering raw lacquer waterborne, successfully constructing a stable oil-in-water (O/W) raw lacquer emulsion system [13]. The core of this research lay in the design and synthesis of a reactive urushiol-based emulsifier (UE): using urushiol, epichlorohydrin, and poly(ethylene glycol) (PEG) as raw materials, an amphiphilic macromolecular emulsifier was synthesized via ring-opening polymerization, in which the hydrophilic segment (PEG) and the hydrophobic anchoring group (urushiol aromatic ring) are highly compatible with the raw lacquer matrix. Using a phase inversion technique, raw lacquer, emulsifier, and water were mixed under high-speed stirring. When the water content reached a critical value, the system spontaneously transformed from a W/O type to an O/W type, forming a stable emulsion with a particle size of less than 400 nm. The resulting waterborne raw lacquer emulsion (RLE) exhibited good storage stability (remained homogeneous without stratification for more than 3 months at room temperature) and retained the excellent characteristics of natural raw lacquer after film formation. After blending with styrene-acrylic emulsion (PSA) or pure acrylic emulsion (PA), the coating film achieved an adhesion grade of 1, a pencil hardness of 3H, a flexibility of 2 mm, while also exhibiting excellent chemical corrosion resistance and heat resistance. This work not only solved the technical bottleneck of rendering raw lacquer waterborne but also achieved the integration of emulsifier and film-forming material through molecular design, providing a replicable paradigm for the waterborne modification of other natural resins.

### 3.3. Biomass Resource Synergistic Composite Modification

Biomass resource synergistic composite modification emphasizes the concept of “derived from nature, superior to nature,” achieving performance complementarity and functional superposition by combining raw lacquer with other natural polymers or biomimetic systems.

In terms of natural polymer composite, Yu Shi et al. [16] reviewed that urushiol in raw lacquer contains a long alkyl side chain and reactive phenolic hydroxyl groups, possessing the potential to interact with various biomacromolecules (such as chitosan, lignin, and tannic acid).

### 3.4. Curing Behavior Regulation

The curing of raw lacquer is essentially an oxidative polymerization reaction of urushiol catalyzed by laccase, a process highly dependent on environmental humidity (typically requiring RH > 70%) and a suitable temperature (25–30 °C). Regulating laccase activity, oxygen diffusion rate, or the molecular arrangement of urushiol through the addition of exogenous additives to optimize drying kinetics and film formation quality has become an important direction in raw lacquer modification. Zheng Binbin systematically investigated the effects of three types of modifiers—inorganic salts, nonionic surfactants, and salicylaldehyde Schiff bases—on the curing behavior of raw lacquer, providing a theoretical basis and technical pathway for precisely controlling the drying process of raw lacquer [17].

#### 3.4.1. Inorganic Salts

Inorganic salts, as inexpensive and readily available additives, can significantly influence the curing behavior of raw lacquer by regulating laccase activity, substrate reaction pathways, and radical generation kinetics. Zheng Binbin et al. systematically investigated the effects of NaCl and Na_2_CO_3_ on the drying process of raw lacquer, revealing their dual “inhibition–activation” regulatory mechanism on curing rate, and proposed for the first time the “ionic microenvironment engineering” strategy [17]. The study found that the single addition of either NaCl or Na_2_CO_3_ significantly delayed the drying of raw lacquer: with the addition of 0.82 wt% NaCl, the surface drying time was extended from 3 h (control group) to 37.5 h, and the through-drying time was extended to 53.5 h; with the addition of 0.82 wt% Na_2_CO_3_, the through-drying time was extended to more than 15 days. Mechanistic analysis indicated that Cl^−^ can competitively coordinate with Cu^2+^ in the active center of laccase, reducing the relative activity of laccase by approximately 35%, thereby inhibiting the oxidative polymerization of urushiol; CO_3_^2−^, on the other hand, forms semiquinone anions by abstracting protons from the phenolic hydroxyl groups of urushiol, hindering electron transfer and radical coupling processes in the laccase catalytic cycle, further inhibiting the polymerization reaction. Further studies showed that by precisely adjusting the compounding ratio of NaCl and Na_2_CO_3_, a “non-monotonic regulation” of curing rate could be achieved: when the two were blended at 0.41 wt%:0.41 wt%, the surface drying time of the lacquer was shortened to 6 h, and the through-drying time was reduced to 9 h, outperforming the effect of single salt addition. Mechanistic analysis indicated that the introduction of Cl^−^ partially competitively inhibits the proton binding of CO_3_^2−^ with urushiol, restoring the laccase catalytic pathway; at the same time, CO_3_^2−^ combines with H^+^ generated in the reaction system to form HCO_3_^−^, reducing the concentration of semiquinone anions and allowing the oxidative polymerization reaction to proceed smoothly [17]. This study proposed for the first time the “ionic microenvironment engineering” strategy, achieving precise control over the drying kinetics of raw lacquer by constructing a multi-ion synergistic system, providing a new approach for developing low-cost, fast-drying natural coatings, while also offering an effective pathway to address the “skin formation” issue caused by persistent laccase activity during raw lacquer storage.

#### 3.4.2. Nonionic Surfactants

Nonionic surfactants have significant application value in improving the wettability, rheology, and emulsion stability of coatings due to their excellent amphiphilicity. Zheng Binbin systematically investigated the effects of glycerol and polyphenolic antioxidants (gallic acid, caffeic acid, cinnamic acid, and ethyl gallate) on the curing behavior of raw lacquer, revealing their dual roles in regulating emulsion structure and modifying color [17]. Experiments show that the addition of glycerol significantly affects the drying performance and rheological behavior of raw lacquer. When the addition ratio is mass of glycerol:mass of refined raw lacquer = 6.5:10, under conditions of 35 °C and 80% relative humidity, the surface drying time of the modified lacquer is shortened to 1 h, and the through drying time is reduced to 3 h and 24 min. The drying rate is approximately three times higher than that of unmodified raw lacquer. Rheological studies indicated that the addition of glycerol increased the apparent viscosity of the modified lacquer from approximately 4 Pa·s to 50 Pa·s, enhanced the thixotropy, and the lacquer exhibited more pronounced pseudoplastic fluid characteristics. This is because the amphiphilic groups of glycerol enhance the stability of the “water-in-oil” emulsion structure of the lacquer, making the droplet size distribution of the dispersed phase more uniform. SEM observation showed that an appropriate amount of glycerol (4:10 to 6.5:10) resulted in a uniform porous structure in the modified lacquer film, while excessive addition (8:10) led to pore reduction and surface densification. Meanwhile, the introduction of polyphenolic antioxidants caused a significant change in the lacquer film color: after immersion–drying–low humidity ventilation treatment, the modified lacquer film changed from traditional black to a matte light yellow, effectively improving the inherent “black color” defect of raw lacquer. However, antioxidants scavenge some of the urushiol quinone radicals, hindering the polymerization reaction and resulting in prolonged drying time. This study clearly pointed out that the application of glycerol and antioxidants in raw lacquer requires precise control of the ratio, with the goals of “emulsion structure stabilization” and “color regulation.” The optimal glycerol addition ratio is 6.5:10, where the lacquer film exhibits rapid drying, a porous structure, and tunable color, providing an experimental basis for the development of functional raw lacquer coatings [17].

#### 3.4.3. Salicylaldehyde Schiff Bases

Traditional raw lacquer driers mostly rely on heavy metal salts (such as cobalt and manganese soaps), which pose risks of toxicity and environmental pollution. The development of efficient, low-toxicity novel drying systems has become a research hotspot. Salicylaldehyde Schiff bases are a class of organic ligands formed by the condensation of salicylaldehyde with amine compounds. Their molecules contain O, N-bidentate chelation sites, enabling the formation of stable complexes with transition metal ions, exhibiting potential catalytic activity. Zheng Binbin introduced such compounds into the raw lacquer system for the first time, synthesizing three salicylaldehyde Schiff base ligands and their copper complexes—N, N-bis-salicylaldehyde-ethylenediamine (SED), N, N-bis-salicylaldehyde-p-phenylenediamine (SPP), and N, N-bis-salicylaldehyde-o-phenylenediamine (SOP)—and systematically explored their effects on the curing behavior of raw lacquer [17]. The results showed that under ambient temperature conditions (25 °C, 80% RH), except for SED, neither the Schiff base ligands nor their copper complexes promoted rapid drying of the lacquer; instead, they partially inactivated laccase, leading to prolonged drying time. For example, after adding 5 wt% SPP-Cu, the surface drying time was extended to 10 h 40 min, and the through-drying time was extended to 21 h 11 min. However, under thermal polymerization conditions at 140 °C, the Schiff base ligands and their copper complexes exhibited significant catalytic effects: the addition of 7 wt% SED shortened the surface drying time to 1 h 01 min and the through-drying time to 2 h 07 min, approximately twice as fast as refined raw lacquer (surface drying 3 h 18 min, through-drying 5 h 52 min); the addition of 5 wt% SPP-Cu also reduced the surface drying time to 1 h 08 min. Mechanistic studies indicated that the Schiff base ligands and their copper complexes affect the process of urushiol dimer or oligomer formation. Infrared spectroscopy showed shifts in the antenna-like absorption peaks in the modified lacquer film, indicating changes in benzene ring coupling and skeletal vibration modes. Thermogravimetric analysis showed that the addition of Schiff bases slightly reduced the thermal stability of the lacquer film but resulted in a denser surface and enhanced hydrophobicity (contact angle increased from 88.1° to 96.3°). This study demonstrates that salicylaldehyde Schiff bases and their copper complexes have application potential as high-temperature thermal polymerization catalysts, providing new ideas for developing low-toxicity, high-efficiency thermal curing systems for raw lacquer. However, their catalytic effect at room temperature is limited, and the addition amount must be strictly controlled to avoid negative impacts on laccase activity. Future work could further improve their catalytic efficiency and applicability by optimizing the molecular structure of Schiff bases (e.g., changing the amine component, introducing functional groups) [17].

## 4. High-Value-Added Applications of Raw Lacquer

### 4.1. Preventive Conservation Materials for Cultural Heritage

In recent years, with the deepening of the concept of intangible cultural heritage protection, especially under the promotion of the “productive protection” model, the application of raw lacquer is no longer limited to traditional lacquerware production but has gradually expanded to the field of preventive conservation of cultural heritage, demonstrating significant high-value-added potential [37,38]. The unique advantages of raw lacquer in cultural heritage conservation stem from its excellent physical and chemical properties: the lacquer film is dense, hard, and abrasion-resistant, and exhibits excellent resistance to various corrosive chemical media [39]. Lacquerware from the Warring States period and Han Dynasty unearthed in archaeological excavations, despite being buried for thousands of years, still has lacquer layers that are as bright as new, and the substrates are preserved from decay due to the protection of the lacquer film, fully confirming the excellent effectiveness of raw lacquer in isolating oxygen, moisture, and microorganisms [37]. These characteristics make it highly suitable for the conservation of cultural heritage objects, environmental control of storage rooms, and surface treatment of exhibition facilities.

In the specific application of preventive conservation, the high added value of raw lacquer is reflected in the following four aspects: First, as a surface sealing material for cultural relics. Raw lacquer can be used for surface reinforcement and sealing of wooden, ceramic, and even some stone cultural relics, preventing temperature and humidity fluctuations, pollutant adhesion, and microbial erosion. Compared with synthetic resins, raw lacquer is non-toxic and harmless, easy to repair after aging, and has better compatibility with cultural relic materials [40]. Second, applied to micro-environment control for cultural relic storage and display. Raw lacquer coatings can be used to produce cultural relic boxes, cabinet liners, and interior surfaces of display cases, utilizing their antibacterial and humidity-regulating properties to create a stable and clean preservation micro-environment. Third, as a new type of protective packaging material. By combining raw lacquer with natural fibers (such as hemp or paper), lightweight, strong, cushioning, and humidity-regulating inner linings for cultural relic transport packaging can be developed, reducing physical and chemical risks during transport. Fourth, biomimetic coating research on raw lacquer provides new ideas for the protection of immovable outdoor cultural relics (such as ancient building wooden components and stone carvings). Through modified raw lacquer, protective coatings with superhydrophobic, self-cleaning, and UV-resistant functions can be prepared to delay weathering processes.

The high-value-added application of raw lacquer not only expands its field of use but also promotes the sustainable utilization of lacquer tree resources and the upgrading of related industrial chains. By introducing raw lacquer into the field of cultural heritage conservation science and technology, not only is the ecological nature and cultural connotation of conservation materials enhanced, but new market demands are also created for the traditional lacquer art industry, achieving a win–win situation of “conservation” and “development” [38]. This application model is a vivid practice of the productive protection concept of intangible cultural heritage—by innovating application scenarios while preserving the authenticity of the core techniques (raw lacquer refining and coating), traditional materials are revitalized in modern scientific and technological systems [41].

It is noteworthy that the raw lacquer species identification technology developed by the Shandong University team also serves the field of cultural heritage conservation [23]. Using a combination of high-performance liquid chromatography (HPLC) and enzyme-linked immunosorbent assay (ELISA), specific antibodies were prepared, and an enzyme-linked immunosorbent quantitative analysis method was established, enabling accurate identification of the raw lacquer species used in lacquerware cultural relics. This provides key technical support for studying the development of ancient lacquer tree cultivation and lacquerware craftsmanship, and also lays a foundation for customizing conservation plans based on the characteristics of raw lacquer from different species [23].

In the future, it is necessary to further strengthen quantitative research on the performance of raw lacquer, the formulation of standardized processes, and interdisciplinary integration with modern conservation science, and to establish technical standards and effect evaluation systems for the application of raw lacquer in the preventive conservation of cultural heritage. At the same time, attention should be paid to the collaboration between inheritors and research institutions, combining traditional experience with modern science and technology, so that raw lacquer, a natural material carrying profound cultural memory, can play a greater and more sustainable role in the mission of safeguarding the common cultural heritage of humanity.

### 4.2. Advanced Functional Coatings and Surface/Interface Engineering

#### 4.2.1. Raw Lacquer Anti-Corrosion Coatings

As a natural anti-corrosion coating, the modern application of raw lacquer should draw on the concept of compounding and synergy from ancient wisdom, achieving performance leaps through material design and interface engineering. In harsh industrial environments (such as offshore platforms, chemical pipelines, and underground facilities), a raw lacquer-based composite coating system can be adopted: through a layered coating process, a high-permeability raw lacquer formulation is used for the bottom layer to ensure tight adhesion to the substrate and pore sealing, while the top layer is formulated as a denser, more weather-resistant lacquer film, constructing a macroscopic functionally graded barrier [31]. At the formulation design level, the ancient “organic–inorganic composite” concept can be simulated by compounding raw lacquer with surface-modified nano-inorganic fillers (such as nano-silica and graphene oxide), replacing traditional cinnabar and ochre. The high barrier properties of nanoparticles effectively delay the permeation of water, oxygen, and corrosive ions. At the same time, flexible natural or synthetic resins (such as tung oil) can be introduced for blending to enhance the flexibility and crack resistance of the coating, ensuring its integrity under temperature changes or mechanical stress. It is worth learning from the emphasis on interfacial stability: by imitating the pretreatment concept of “pre-mixing cinnabar with tung oil,” fillers are organically coated or treated with coupling agents to strengthen their interfacial bonding with the raw lacquer matrix, avoiding corrosion channels caused by interfacial defects. In addition, for long-term protection, the potential for “self-sealing” pores through the reaction of the coating with specific ions in the environment to form fillers can be explored, further enhancing its durability. In recent years, researchers have further compounded urushiol with various nanomaterials to construct advanced anti-corrosion systems with synergistic enhancement effects. For example, compounding urushiol-formaldehyde polymer with graphene oxide and multi-walled carbon nanotubes can significantly reduce the corrosion rate of metal substrates, with a protection efficiency as high as 99.70%, demonstrating great potential for application in harsh marine environments; by introducing components such as montmorillonite and rosin-modified cuprous oxide compounded with urushiol-titanium polymer, the density and adhesion of the coating can be effectively improved, maintaining stable anti-corrosion performance even after long-term immersion [42]. The above research provides a solid technical foundation for the development of high-performance environmentally friendly coatings suitable for heavy-duty anti-corrosion fields (such as ships, chemical equipment, and marine engineering).

The exceptional long-term durability of raw lacquer is further supported by archaeological evidence. Table 3 compares the anti-corrosion performance of raw lacquer with modern synthetic coatings based on both archaeological findings and contemporary studies. As shown in Table 3, raw lacquer coatings have demonstrated a proven service life of nearly 2000 years under burial conditions, as evidenced by the well-preserved lacquer films from the Noin-Ula burial complex in Mongolia, where the original wooden substrates had completely decayed while the lacquer coatings remained intact [42]. This millennial-scale durability is unmatched by any modern synthetic coating system, which typically exhibits effective service lives of 20–50 years. While synthetic coatings offer advantages in terms of faster curing and lower cost, the unparalleled longevity of raw lacquer positions it as a uniquely valuable material for long-term heritage protection and infrastructure applications where maintenance is difficult or costly.

#### 4.2.2. Superhydrophobic Coatings

**Figure 9:**
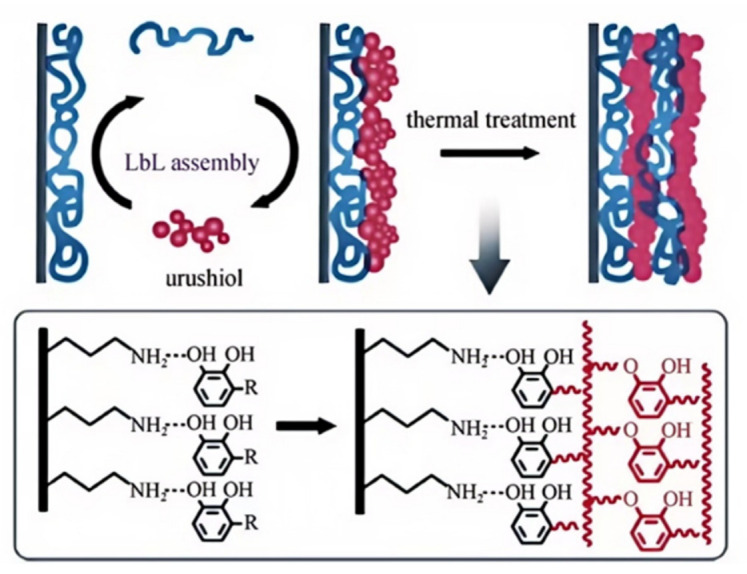
Multilayer nano-film prepared by LbL process and post-treatment [41].

The application of raw lacquer as a hydrophobic coating can be achieved through a simple and efficient surface morphology control technology. Yuansong Ye et al. proposed a particle template method: using inorganic salt particles with a specific particle size as a template, they were pressed into the surface during the high-temperature pre-curing stage of raw lacquer; after the lacquer film was fully cured, the template was removed, thereby constructing a micro-nano composite porous structure on the surface [30]. This process does not alter the chemical composition of raw lacquer itself but increases its water contact angle from the intrinsic approximately 78° to 153.5°, achieving a superhydrophobic state (Figure 9). The modified raw lacquer film exhibited excellent self-cleaning, waterproofing, and anti-fouling functions, and maintained good hydrophobic stability even after long-term outdoor exposure. This technique is easy to operate, low-cost, and suitable for large-area preparation, providing a feasible solution for the large-scale functional application of raw lacquer, a traditional natural material, in fields such as modern building exterior walls, outdoor facilities, and anti-fouling coatings. In addition to the particle template method, researchers have developed other pathways to impart super-hydrophobicity to urushiol surfaces. For example, utilizing the chelation effect between the catechol group of urushiol and copper ions, urushiol–copper nanoparticles can be prepared, and then a superhydrophobic film with micro/nano hierarchical structures can be obtained through layer-by-layer assembly technology, achieving a water contact angle of up to 152° [42]; furthermore, the long alkyl side chain and unsaturated double bonds of urushiol itself enable it to form a hydrophobic coating through self-polymerization on the surface of porous materials (such as sponges) upon heat treatment, demonstrating efficient application potential in the fields of oil–water separation and oil absorption. These methods provide diverse technical options for the development of novel, multifunctional superhydrophobic engineering materials.

To quantitatively demonstrate the advantages of the modified raw lacquer superhydrophobic coating, Table 4 compares its key performance metrics with those of commercial synthetic coatings.

As shown in Table 4, the modified raw lacquer achieves a water contact angle of 153.5°, which is significantly higher than conventional epoxy (70–80°), polyurethane (80–100°), and even fluoropolymer coatings (110–120°) [30]. This super-hydrophobicity is achieved through surface microstructure engineering (particle template method) without altering the chemical composition of the lacquer. The modified surface also maintains good durability under outdoor conditions, with the water contact angle decreasing only from 153.5° to 150.8° after 30 days of outdoor exposure. This comparison suggests that modified raw lacquer has the potential to compete with or even surpass commercial synthetic coatings in superhydrophobic applications, particularly where self-cleaning and water repellency are required.

#### 4.2.3. Flame-Retardant Coatings

The key to the application of raw lacquer as a flame-retardant coating lies in overcoming its inherent flammability through functional additives, thereby expanding its practicality in fields with high fire safety requirements. Shiu et al. introduced a phosphorus–nitrogen–silicon composite intumescent flame retardant (IFR) into the raw lacquer system, successfully preparing a composite coating that combines excellent fire performance with the inherent advantages of raw lacquer [43]. Specifically, after blending an IFR with a specific ratio (FR-1:FR-2:diatomaceous earth weight ratio = 56:16:28) at an addition level of 30 wt% with raw lacquer and curing, the resulting composite coating exhibited a limiting oxygen index (LOI) of 30.2%, achieving the highest V-0 flame retardancy rating according to the UL-94 standard [44], and demonstrated rapid self-extinguishing behavior without dripping in vertical burning tests. Mechanistic studies indicated that this flame-retardant coating forms a dense, intumescent char layer at high temperatures, effectively isolating heat and oxygen, and significantly reducing the heat release rate and total heat release [43]. Meanwhile, the modified coating maintained the traditional high adhesion, good gloss, and outstanding acid corrosion resistance of raw lacquer, and its mechanical properties and alkali resistance were also somewhat improved. This technology provides a feasible functional coating solution for raw lacquer, an environmentally friendly material, in fields such as building interiors, furniture coatings, cultural relic protection, and marine applications where both fire protection and durability are required.

### 4.3. Biomedical and Environmental Materials

#### 4.3.1. Hemostatic and Antibacterial Materials

Natural plant phenols generally possess antibacterial properties, and their specific structures, after derivatization treatment, can effectively inhibit the growth of various fungi and bacteria [42]. In recent years, significant progress has been made in the research of urushiol-based antibacterial and hemostatic functional materials. Qi et al. used in situ polymerization technology to prepare essential oil-loaded microcapsules (E-UMs) with urushiol microcapsules (UMs) as the shell and essential oil (EO) as the core. The results showed that the mass fraction of EO in E-UMs was as high as 30%, and the release rate was less than 1.87% after treatment at 100 °C for 6 h. This coating exhibited good efficacy against drug-resistant *Helicobacter pylori* and *Staphylococcus aureus*. Zheng et al. synthesized polymerized urushiol (PUL) encapsulating silver nanoparticles (AgNPs) via a simple one-pot method. The combination of AgNPs and PUL enabled the stable release of Ag^+^, and the coating containing 0.1% AgNPs achieved an antibacterial rate of 100%. Chen et al. synthesized a urushiol benzoxazine copper polymer (UBCP), which can be cured at room temperature to form a film with strong adhesion, a smooth and dense surface, and exhibited nearly 100% antibacterial performance against both *Escherichia coli* and *Staphylococcus aureus*, as well as over 99% inhibition of algal activity, effectively preventing microbial adhesion.

In terms of hemostatic materials, Liu Shuqiong et al. first prepared a chitosan nanofiber membrane via thermally induced phase separation, then introduced reactive free radicals using low-temperature plasma irradiation to graft urushiol onto the fiber membrane, and finally compounded it with graphene oxide followed by glutaraldehyde crosslinking to obtain a chitosan-grafted urushiol/graphene oxide composite material. This material exhibited efficient and rapid hemostatic capability [42]. In addition, Chen et al. synthesized a urushiol-functionalized mesoporous silica nanomaterial with a core–shell structure (MSN@U). The hydrophilic catechol groups and long hydrophobic alkyl chains in urushiol enable it to self-assemble at the blood–air interface, forming a nanomaterial with both good hemostatic effect and blood compatibility. The above studies indicate that urushiol-based materials have broad application prospects in the fields of wound repair, antibacterial coatings, and anti-biofouling.

#### 4.3.2. Drug Controlled-Release Carriers

**Figure 10:**
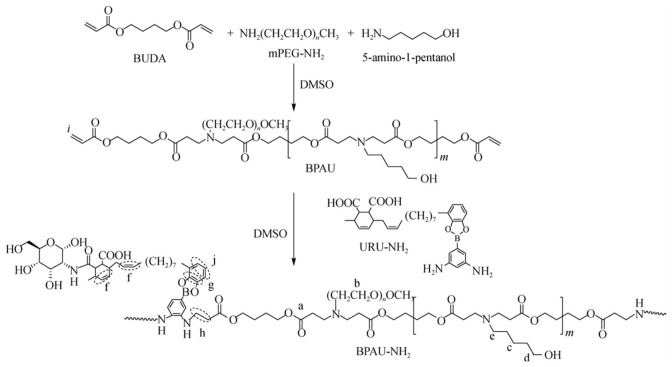
Synthesis route of pH-responsive micelle BPAU-NH_2_-Gal [45].

Urushiol has unique application value as a drug controlled-release carrier, particularly for targeted antitumor therapy. Qi Zhiwen et al. successfully constructed a pH-responsive nanoscale drug-loaded micelle, BPAU-NH_2_-Gal (Figure 10), using urushiol borate derivative (URU-NH_2_) as the hydrophobic core combined with galactose (Gal) as the targeting recognition unit [45]. The micelle had a particle size of approximately 195 nm, a positive surface charge (Zeta potential of +29.7 mV), and could effectively encapsulate the hydrophobic antitumor drug paclitaxel, with an encapsulation efficiency as high as 92.51% and a drug loading capacity of 31.76%. Its most outstanding application characteristic lies in its intelligent drug release capability: under simulated tumor acidic microenvironment conditions (pH 4.5), the drug was released rapidly, achieving a cumulative release rate of 50.00% at 90 h; under normal physiological conditions (pH 7.4), the release was slow, with a cumulative release rate of only 9.00%, demonstrating significant pH-responsive release behavior, thereby enabling selective drug release at the tumor site. In addition, this drug-loaded micelle exhibited low toxicity toward human normal liver cells (LO2), showed good biocompatibility, and could be efficiently taken up by human hepatocellular carcinoma cells (HepG2). This research provides a feasible solution for developing intelligent targeted drug delivery systems based on natural urushiol, particularly demonstrating good application prospects in improving the solubility of hydrophobic drugs, reducing systemic toxicity, and achieving tumor-specific therapy.

#### 4.3.3. Water Treatment Adsorption Materials

Urushiol, as a green reducing agent and modifier, has shown significant potential in the development of efficient, multifunctional, and environmentally friendly water treatment adsorption materials [46]. Studies have shown that urushiol can be used to prepare well-dispersed urushiol-modified reduced graphene oxide (U-rGO), thereby enhancing the performance of urushiol composite films in chemical-resistant and anti-corrosion coatings [46]. The urushiol-modified graphene/Fe_3_O_4_ composite (U-Fe_3_O_4_-rGO) synthesized via a one-step method can efficiently catalyze the degradation of dye wastewater—achieving a decolorization rate of 97.8% for rhodamine B within 20 min—while also adsorbing heavy metal ions (e.g., an adsorption capacity of 158.9 mg/g for Cr(VI)), and is easily recoverable by magnetic separation with excellent cycling performance [46]. In addition, graphene/polyurushiol aerogels constructed with urushiol exhibit a porous ultralight structure and good mechanical stability, making them suitable for efficient adsorption of water pollutants and energy storage applications [46]. The above studies fully demonstrate the unique advantages of urushiol in the field of environmental remediation materials: on the one hand, the catechol structure in the urushiol molecule can serve as a green reducing agent, enabling mild and efficient reduction in functional materials such as graphene; on the other hand, the long alkyl side chain of urushiol imparts good hydrophobicity and affinity for organic pollutants to the composite material, expanding its application potential in fields such as oil–water separation. These studies provide new ideas for the development of high-performance environmental functional materials based on natural products.

#### 4.3.4. Safety and Toxicity Considerations

Urushiol, the primary active component of raw lacquer, is well known as a potent contact allergen, capable of inducing severe allergic contact dermatitis in sensitized individuals. This inherent toxicity poses significant safety concerns for the practical application of raw lacquer-based materials, particularly in waterborne coatings, drug delivery systems, and biomedical devices where direct or indirect human contact is anticipated. Among the studies cited in this review, the majority focused on performance optimization (e.g., drying time, mechanical properties, corrosion resistance) without systematic assessment of cytotoxicity, dermal irritation, or environmental impact. For instance, studies on aminoanthraquinone-grafted urushiol [10] and allyl-modified raw lacquer [15] did not include any biocompatibility evaluation. In the biomedical domain, while Qi et al. [45] reported low toxicity of BPAU-NH_2_-Gal micelles toward human normal liver cells (LO2), no standardized cytotoxicity assays (e.g., ISO 10993-5 [47]) or skin sensitization tests (e.g., local lymph node assay) were performed. Similarly, the hemostatic materials developed by Liu et al. [42] and the urushiol-functionalized mesoporous silica nanomaterials [42] lacked comprehensive in vivo toxicity or immunogenicity assessments.

To bridge this gap, future research on modified raw lacquer should adhere to established safety evaluation protocols. For biomedical applications, a minimum dataset should include: (1) in vitro cytotoxicity using relevant cell lines (e.g., L929 fibroblasts, HaCaT keratinocytes) according to ISO 10993-5; (2) skin irritation and sensitization tests using reconstructed human epidermis (RhE) models or animal studies; (3) assessment of residual monomer content (especially unreacted urushiol) after curing; and (4) evaluation of leachables and degradation products under physiological conditions. For environmental applications, biodegradability and ecotoxicity assays (e.g., using Daphnia magna or Vibrio fischeri) are recommended. Only with such rigorous safety validation can modified raw lacquer be considered a viable candidate for consumer-facing or medical-use products.

### 4.4. Smart Responsive and Flexible Electronic Materials

Smart responsive materials can produce controllable physical or chemical responses to external stimuli (such as light, heat, electricity, pH, etc.) and represent a frontier direction in current functional materials research. As a natural polymer material, raw lacquer has achieved important breakthroughs in recent years in the exploration of applications in smart responsive and flexible electronics. Researchers have utilized polymer nanoparticles formed by the coordination of urushiol with metal ions (such as iron ions) as functional fillers incorporated into matrices such as thermoplastic polyurethane, successfully developing shape memory composites with excellent photothermal response properties [48]. The core value of this type of material lies in its expansion of practical application scenarios: it enables remote, non-contact deformation control through near-infrared (NIR) light, avoiding the dependence on complex wiring or direct heating required by traditional thermally or electrically driven actuation methods, offering significant advantages in microdevices, flexible electronics, and smart structures. For example, the research team has constructed a prototype light-controlled switch device based on this composite material—when irradiated with NIR light, the material rapidly heats up and triggers shape recovery, thereby automatically closing the circuit to illuminate an LED bulb, demonstrating its feasibility in light-driven actuators and smart sensing systems.

This urushiol-based composite material not only exhibits high shape fixation and recovery ratios of up to 99.44% but also demonstrates good mechanical strength and processability, making it suitable for engineering environments with high requirements for precision and reliability (Table 5). More importantly, its shape memory behavior can be precisely regulated over a wide temperature range from 37 °C to 80 °C, supporting multiple shape programming and enabling the realization of complex action sequences. This characteristic is particularly critical in biomedical engineering, such as for self-expanding stents in minimally invasive surgery, targeted drug delivery carriers, or deformable implantable devices, where materials are often required to respond near body temperature and precisely complete predefined deformations. At the same time, the urushiol–iron nanostructure itself possesses efficient photothermal conversion capability, which not only drives deformation under light irradiation but also generates localized high temperatures, enabling rapid killing of common pathogens such as *Escherichia coli*. This dual “deformation + bactericidal” function gives this material unique potential in medical protective equipment such as smart dressings for hospitals, self-cleaning catheters, and protective face masks.

Since urushiol is derived from natural raw lacquer and possesses good biocompatibility and environmental friendliness, compared with traditional petroleum-based smart materials, it also holds strategic significance in terms of sustainable development and green manufacturing. Currently, this type of composite material has been validated at the laboratory scale for its application potential in cutting-edge fields such as soft robotics, reconfigurable antennas, and adaptive optical devices. In the future, with the optimization of processing techniques and the maturation of large-scale preparation technologies, urushiol-based shape memory composites are expected to achieve practical applications in more high-value-added scenarios such as smart textiles, aerospace deployable structures, and human–machine interaction interfaces, promoting the transformation and upgrading of natural polymer materials from traditional coatings to high-end functional materials.

## 5. Challenges and Future Perspectives

### 5.1. Fundamental Scientific Issues and Interdisciplinary Innovation

Current research on raw lacquer suffers from a fragmented academic landscape and significant disciplinary imbalance. Bibliometric analysis reveals that existing studies are predominantly concentrated in fine arts (approximately 40.31%), while disciplines such as chemistry, materials science, and economics remain severely underrepresented [48]. Collaboration networks among researchers and institutions are loose, with network densities of only 0.0029 for authors and 0.0013 for institutions, indicating that the field has not yet formed a highly integrated collaboration network [49].

Future research should prioritize interdisciplinary collaboration to elucidate the fundamental structure–property relationships of raw lacquer components [50]. Systematic studies on lacquer germplasm resources using modern analytical techniques are needed to establish reliable correlations between chemical composition and macroscopic film properties [51]. Understanding the genetic basis of compositional differences between wild and domesticated lacquer varieties will facilitate targeted breeding of high-performance lacquer trees [23].

### 5.2. Green Modification Strategies and Sustainable Industrialization

As a renewable resource, the whole-life-cycle greenness of raw lacquer is a core direction for future development [52]. Waterborne modification represents a particularly promising green strategy, as it preserves the eco-friendly characteristics of raw lacquer while addressing issues of high viscosity and difficult application. Studies have demonstrated that stable oil-in-water raw lacquer emulsions can be prepared using composite emulsifiers, successfully reducing curing time from 4–5 days to 3 days while maintaining excellent mechanical properties and chemical resistance [53]. However, current waterborne raw lacquer systems still face technical bottlenecks, including poor long-term storage stability and insufficient curing rates under ambient conditions [13]. Future research should focus on developing more efficient and safer emulsifiers, optimizing curing catalytic systems, and exploring biomimetic emulsion systems [54]. The introduction of UV-curing technology and nano-modification has also shown promise for improving drying speed, mechanical properties, and UV aging resistance [55].

None of the cited studies conducted a life cycle assessment (LCA) or carbon footprint calculation for the described modification strategies. Several widely used modifiers raise sustainability concerns: silane coupling agents are petroleum-derived; epichlorohydrin is a probable human carcinogen (Group 2A by IARC); and functional nanomaterials require energy-intensive production. Even bio-based modifiers like CNF require chemical pretreatments that generate wastewater. Future research should prioritize comparative LCA studies, develop fully bio-based and non-toxic additives, and establish green chemistry metrics to guide sustainable innovation. Only by addressing these issues can modified raw lacquer legitimately claim to be a sustainable alternative to synthetic petroleum-based coatings.

### 5.3. Multifunctional Integration and Smart Adaptive Systems

Developing raw lacquer from a single-function coating material into multifunctional integrated systems is an important pathway to enhance its added value [55]. By incorporating functional nanofillers or stimulus-responsive elements, raw lacquer-based materials could achieve combined anti-corrosion, superhydrophobic, antibacterial, or self-healing properties [27].

Smart adaptive raw lacquer materials represent another frontier direction. By introducing thermo-responsive, photo-responsive, or pH-responsive units, raw lacquer materials could autonomously adjust their performance in response to environmental changes. Such smart materials hold significant application potential in high-tech fields including cultural heritage conservation, aerospace components, and biomedical devices [56].

### 5.4. Standardization and Big Data-Driven Research Paradigms

The field of raw lacquer research currently lacks unified performance evaluation standards and material characterization specifications, making it difficult to directly compare research results across different studies. The interpretation of historical Chinese texts on lacquer technology faces significant challenges, including changes in terminology and measurement units across different dynasties. For example, the length unit “chi” varied from approximately 19.91 cm in the Zhou Dynasty to 32.0 cm in the Qing Dynasty. These challenges must be addressed through systematic efforts to digitize and standardize historical knowledge [57].

At the same time, introducing big data and artificial intelligence technologies to construct composition–structure–property databases for raw lacquer is expected to accelerate the design of new materials and reduce trial-and-error costs [55].

## 6. Conclusions and Outlook

Raw lacquer, as a natural material with a thousand-year tradition, is being revitalized through scientific and technological innovation. This paper systematically reviews the recent progress in the modification and application of raw lacquer, with particular emphasis on frontier directions such as nanocomposite modification, interface engineering, and biomass synergistic composite modification. Research has shown that through multi-scale structural regulation and functional integration, raw lacquer has expanded from traditional coating applications to emerging fields such as cultural heritage conservation, advanced functional coatings, biomedical materials, and smart responsive systems.

Future research on raw lacquer will place greater emphasis on interdisciplinary integration, balancing fundamental research with technological innovation, and pursuing a development path that unifies green sustainability with high performance. As the understanding of the relationship between the microstructure and properties of raw lacquer deepens, and with continuous innovation in modification technologies, this ancient natural material is expected to play an increasingly important role in cutting-edge fields such as high-end manufacturing, new energy, and biomedicine, achieving a magnificent transformation from the traditional “king of coatings” to a modern “multifunctional smart material.”

As an excellent representative of green sustainable materials, the research and development of raw lacquer not only hold scientific significance but also align closely with the national green development strategy. Exploring the potential of this traditional material through scientific and technological innovation is not only an inheritance of China’s excellent traditional culture but also a concrete practice in promoting the sustainable development of the materials industry, and will undoubtedly contribute to the construction of a resource-conserving and environment-friendly society.

## Figures and Tables

**Table 1:** Experimental record of each property of lacquer films.

SiO_2_ Content (wt%)	Pencil Hardness	Adhesion Grade	Glossiness of Lacquer Film (%)
0	3H		~70
0.25	4H	1	
0.50	4H	1	
>0.50	3H	2	~40

**Table 2:** Physical properties of urushiol film and 2-aminoanthraquinone-urushiol modified coating film.

Performance	Urushiol Film	2-Aminoanthraquinone-Urushiol Modified Coating Film
Surface drying time/min	240	140
Curing time/h	24	16
Hardness	1H	6H
Impact resistance/cm	18	56
Flexibility/mm	16	4
Adhesion/grade	5	3
Glossiness/%	118	69
Roughness/μm	0.003	0.2

**Table 3:** Comparison of anti-corrosion and durability performance of raw lacquer versus commercial synthetic coatings.

Property	Raw Lacquer (Archaeological Evidence)	Modern Epoxy	Modern Polyurethane	Modern Fluoropolymer
Proven service life	Up to ~2000 years	20–50 years (estimated)	15–30 years (estimated)	30–50 years (estimated)
Substrate protection under burial	Complete (wood decayed, lacquer remained)	Not applicable (no archaeological data)	Not applicable	Not applicable
Resistance to environmental factors	High (soil, moisture, microorganisms)	High	High	Excellent
Natural antimicrobial properties	Yes (urushiol)	No	No	No
Green/renewable	Yes	No	No	No

**Table 4:** Comparison of superhydrophobic modified raw lacquer with commercial synthetic coatings.

Property	Modified Raw Lacquer	Epoxy Coating	Polyurethane Coating	Fluoropolymer Coating
Water contact angle	153.5° [30]	70–80°	80–100°	110–120°
Surface wettability	Super-hydrophobic	Hydrophilic to slightly hydrophobic	Hydrophobic	Hydrophobic
Self-cleaning ability	Yes	No	Limited	Yes
Surface structure	Micro/submicron porous	Smooth	Smooth or textured	Smooth
Preparation method	Particle template (simple, low-cost)	Spray/brush	Spray/brush	Spray
Durability (30 days outdoor)	153.5°→150.8° (minimal loss)	May yellow	Good	Excellent
Green/renewable	Yes	No	No	No

**Table 5:** Shape memory fixation rate and recovery rate of PU2, PU2-UFe0.3, PU2-UFe0.6, PU2-UFe0.9, PU2-UFe1.2.

Performance	PU2	PU2-UFe0.3	PU2-UFe0.6	PU2-UFe0.9	PU2-UFe1.2
θ1	81°	89°	86°	87°	88°
180−θ2	160°	179°	176°	178°	148°
Shape Fixity Ratio (Rf)	90.00%	98.89%	95.56%	96.67%	97.78%
Shape Recovery Ratio (Rs)	88.89%	99.44%	97.78%	97.22%	82.22%

## Data Availability

The original contributions presented in this study are included in the article. Further inquiries can be directed to the corresponding author.
